# Issues and special features of animal health research

**DOI:** 10.1186/1297-9716-42-96

**Published:** 2011-08-24

**Authors:** Christian Ducrot, Bertrand Bed'Hom, Vincent Béringue, Jean-Baptiste Coulon, Christine Fourichon, Jean-Luc Guérin, Stéphane Krebs, Pascal Rainard, Isabelle Schwartz-Cornil, Didier Torny, Muriel Vayssier-Taussat, Stephan Zientara, Etienne Zundel, Thierry Pineau

**Affiliations:** 1 INRA, UR346 Epidémiologie animale, 63122 Saint Genès Champanelle, France; 2 INRA, UMR1313 Génétique Animale et Biologie Intégrative, 78352 Jouy-en-Josas Cedex, France; 3 INRA, UR892 Virologie et Immunologie Moléculaires, 78352 Jouy-en-Josas, France; 4 INRA, Département PHASE, 63122 Saint Genès Champanelle, France; 5 ONIRIS-INRA, UMR1300 Bioagression, Épidémiologie et Analyse de risque, Atlanpole La Chantrerie, BP 40706, 44307 Nantes Cedex 3, France; 6 ENVT-INRA, UMR1225 IHAP Interactions hôtes-agents pathogènes, 23 chemin des Capelles, 31076 Toulouse Cedex, France; 7 INRA, UR1282 IASP Infectiologie animale et santé publique, 37380 Nouzilly, France; 8 INRA, UMR1323 RiTME, 65 Boulevard de Brandebourg, 94205 Ivry-sur-Seine, France; 9 INRA, USC Bartonella et Tiques, ANSES, 23 Avenue du Général de Gaulle, F-94700 Maisons-Alfort, France; 10 ENVA-ANSES-INRA, UMR1161 Virologie, 7 avenue du Général de Gaulle, 94704 Maisons Alfort Cedex, France; 11 INRA, Département de santé animale, 37380 Nouzilly, France; 12 INRA, Département de santé animale, BP 93173, 31027 Toulouse Cedex 3, France

## Abstract

In the rapidly changing context of research on animal health, INRA launched a collective discussion on the challenges facing the field, its distinguishing features, and synergies with biomedical research. As has been declared forcibly by the heads of WHO, FAO and OIE, the challenges facing animal health, beyond diseases transmissible to humans, are critically important and involve food security, agriculture economics, and the ensemble of economic activities associated with agriculture. There are in addition issues related to public health (zoonoses, xenobiotics, antimicrobial resistance), the environment, and animal welfare.

Animal health research is distinguished by particular methodologies and scientific questions that stem from the specific biological features of domestic species and from animal husbandry practices. It generally does not explore the same scientific questions as research on human biology, even when the same pathogens are being studied, and the discipline is rooted in a very specific agricultural and economic context.

Generic and methodological synergies nevertheless exist with biomedical research, particularly with regard to tools and biological models. Certain domestic species furthermore present more functional similarities with humans than laboratory rodents.

The singularity of animal health research in relation to biomedical research should be taken into account in the organization, evaluation, and funding of the field through a policy that clearly recognizes the specific issues at stake. At the same time, the *One Health *approach should facilitate closer collaboration between biomedical and animal health research at the level of research teams and programmes.

## Table of contents

1. Introduction

2. Issues and special features of animal health research

2.1. Animal health and veterinary public health

2.2. Issues at stake in animal health

2.3. Importance of diseases, prioritization of issues at stake

2.3.1. Special features of diseases according to the types of animals

2.3.2. Prioritization of issues at stake

2.3.3. Issues at stake in animal health research

3. Special features of animal health research

3.1. Distinguishing features of the objectives, methods, and biological models

3.2. Special features of scientific questioning

3.3. Generic and methodological areas of convergence with human health

4. Relationships between animal health and human health research

4.1. Domestic animal models for human targeted research

4.2. Funding and evaluation of research

4.3. Parallels between research, surveillance of diseases and the pharmaceutical industry

4.3.1. Surveillance and control of diseases

4.3.2. Pharmaceutical industry

4.4. The "One world, One Health" approach

5. Conclusion

Competing interests

Authors' contributions

Acknowledgements

References

## 1. Introduction

Understanding of animal health research, and the expectations of donors and research organizations, is changing. A growing number of actors consider such research from the limited perspective of the dangers and risks directly posed to human health by traditional and emerging animal diseases. Some furthermore consider health as an asset shared by all species, animal and human, that would be guaranteed by a single medicine guided by biomedical research. In this evolving context, a collective discussion on the special features of animal health research, the issues at stake and the specific contributions such research can provide to generic health research was deemed necessary. This article summarizes the results of this discussion, addressing the issues at stake at the global level. Presented in three sections, the first describes the challenges facing animal health and research on animal health, the majority of which are not related to zoonotic diseases. The second section describes the distinguishing features of animal health research that are related to scientific constraints, the manner by which the discipline is grounded in an agricultural and economic context, and the perspectives from which scientific questions are posed. The third section addresses the relationships between animal health and biomedical research. The conclusion proposes changes that would permit research to be adapted to the special features of the field while at the same time favouring partnerships with research on human health. This discussion deliberately was limited to livestock; pets and wild animals only are mentioned for purposes of comparison.

## 2. Issues and special features of animal health research

### 2.1. Animal health and veterinary public health

In animals, health may be defined as the absence of disease or the normal functioning of an organism and normal behaviour based on the observation of a certain number of individuals that determine the standard and thus health [1]. In production sectors, health also may be defined as the state allowing the highest productivity. However, this narrow definition often is enriched by the concept of a balance between the animal and its environment, and of the animal's physical welfare. This broader definition undoubtedly is linked to changes observed in the field of veterinary medicine, which is focussing increasingly on prevention rather than cure, and which takes the animal's environment into fuller account [2].

Animal diseases may be organized schematically into three categories. **Multifactorial diseases **are provoked by a set of risk factors linked in particular to livestock management, with at times the participation of pathogens widespread in livestock. Known as "production diseases", multifactorial diseases are present on a large majority of livestock farms with highly variable frequencies. The major **epidemic diseases **are highly contagious and impact livestock heavily (for example, foot-and-mouth disease, swine fever, highly pathogenic avian influenza); the challenge is to eradicate such diseases from a territory when possible, and their appearence in a totally susceptible population can have extensive health and economic consequences. Other **transmissible infectious diseases **are less contagious or have slighter impacts, and frequently are present in populations in an endemic manner. Among transmissible diseases are zoonotic diseases, which are those that can be transmitted to humans. Animals also may be healthy carriers of agents that are pathogenic for humans but which do not affect the health of the animal (for example, *Salmonella *and *Campylobacter*).

In response to these challenges, and picking up on a framework produced by international bodies (World Health Organization (WHO), Food and Agriculture Organization of the United Nations (FAO), World organization for Animal Health (OIE)), WHO [3] currently defines veterinary public health as "*the sum of all contributions to the physical, mental and social well-being of humans through an understanding and application of veterinary science*". In an editorial of the OIE bulletin [4], The Veterinary Services are stated as the "*key players in the prevention and control of animal diseases and in the improvement of food security, nutrition, food safety, veterinary public health and market access for animals and products*". Veterinary public health activities thus include the control of animal diseases that have a direct impact on human health due their zoonotic character, as well as the control of all non-transmissible animal diseases capable of causing important production losses (safety of animal product supply) and disrupting markets (animal and products of animal origin).

### 2.2. Issues at stake in animal health

There are four types of issues at stake in the field of animal health:

1/ **Economic issues **for a range of diseases that impact the economic viability of livestock farms (notably livestock diseases and endemic diseases that lead to production losses, prevention or treatment costs, disruption of the farm or the work of the livestock farmer) and animal production sectors (notably epidemic diseases due to their effect on production, the impact of health regulations on markets, and impediments to trade). In industrialized countries, these diseases weigh heavily on the overall economic competiveness of livestock farms, businesses, and animal production sectors. In developing countries, there are the added risks of food scarcity, capital dilution (insofar as cattle constitute standing capital, the only form of savings and social security for many people), and the loss of draught and labor power (leading to a reduction in overall agricultural efficiency).

2/ **Public health issues**, which concern three domains: zoonoses, infectious or parasitic diseases transmissible from animals to humans, whether contagious (for example, tuberculosis, brucellosis, certain influenza viruses), vectorial (West Nile disease, Rift Valley fever, Lyme disease), or food-borne (BSE, toxic food poisoning); resistance to antibiotics; and traces of medicine in animal products.

3/ **Environmental issues **related to the impact of agriculture; this involves the dumping of xenobiotics into the environment (medicine residues), the spread of resistance to antibiotics, and infectious diseases that can be transmitted between domestic and wild animals (such as bovine tuberculosis detected in wildlife).

4/ **Animal welfare issues**, which are related closely to changes of regulations in this domain. Diseases induce suffering and pain, the absence of which is one of the criteria chosen for recently proposed animal welfare evaluation tools [5].

In a recent report on the state of food and agriculture in the world focusing on livestock, the FAO [6] summarizes these different issues at stake as: *"Animal diseases, and a lack of adequate food hygiene resulting in foodborne illnesses, are a problem for everyone because they can threaten human health, disrupt markets and trade, reduce productivity and deepen poverty. Improving the management of livestock with a view to preventing and controlling disease can provide significant economic, social, and human health benefits for the poor and for society at large"*. Among the report's four key messages, it is noted that, "*Livestock diseases pose systemic risks that must be addressed*."

For all of these diseases, while the issues at stake primarily concern agricultural farms, associated economic sectors also are involved: live animals, products of animal origin, agricultural inputs and services. Consumers and citizens are all concerned, as much by quantitative and qualitative food security as by public health. Livestock and agro-food sectors play a central role in industrialized countries, reaching 53% of the gross domestic product [7] (food safety, extensive economic activities linked to supplying the livestock sector which include the pharmaceutical industry, and the valorization and trade of agricultural products and food that often are very technologically advanced), as in developing countries (subsistence agriculture, food security, intake of quality protein). The economic issues involved in animal health, without even mentioning the risks of bioterrorism, therefore represent **critical strategic challenges**, even if they receive less media coverage than public health issues.

Furthermore, these **different types of issues are not independent of each other**. For example, the risk of the presence of medicine residues in animal products, as well as the risk of antibiotic resistance coming from the animal world, are both public health issues, and are both directly correlated to the frequency of enzootic diseases impacting the economic equilibrium of animal production chains; they thus pertain above all to the economic stakes involved in ensuring animal health.

### 2.3. Importance of diseases, prioritization of issues at stake

#### 2.3.1. Special features of diseases according to the types of animals

For production animals, infectious and parasitic diseases predominate, even if metabolic and degenerative disorders naturally exist that most frequently are related to an insufficient control of production systems. In contrast, household pets and sports animals present a pathological profile very similar to humans (endocrinian disorders, cancers, degenerative neuro and osteoarticular diseases, obesity, aging). This leads to a more reduced presence of infectious and parasitic pathologies in favour of internal medicine, cancerology, and endocrinology, although antibiotic and anti-parasite medicines and vaccines together account for 75% of the consumption of medicine by pets. Lastly, non-captive wildlife constitutes a relatively new subject of animal health research, principally concerning major epidemiological reservoirs of potentially zoonotic agents (for example, bat lyssavirus and avian influenza) and sentinels of contamination and toxicologic pollution of the environment.

#### 2.3.2. Prioritization of issues at stake

It is difficult to arrange the different challenges presented by animal diseases and their control into an order of priority. There are several ways to assess the importance of animal diseases. The first is to estimate their **impact on zootechnical and economic performance**. The average mortality rates of animals in Western European livestock systems can be significant for certain age groups, and may reach high levels in herds when pathology is poorly controlled. For example, the mortality of calves before weaning is on average 12%, that of dairy cows 3%, that of piglets before weaning 20% (including stillborns), with another 7% loss between the weaning and slaughter of pigs. The various costs of controlling disease are added to those of mortality. The current economic impact of mastitis in dairy cows in France may be assessed at 350 million €/year, principally due to reductions in productivity and longevity, reduced sale prices of milk and the costs of prevention measures and treatment. In poultry, coccidioses have a major impact; based on a British model [8], their global economic impact is estimated at over two billion dollars, principally due to their impact on production and feed efficiency. In the case of endemic diseases, economic losses remain usually limited in each farm, but the global economic impact is high due to the large number of farms affected [9]. The probability of epidemic diseases is lower but when present, they may induce very severe losses [10], even beyond the agronomic and agri food sectors.

OIE's list of notifiable diseases [11] includes infectious transmissible diseases deemed to be most damaging at the international level from an economic and public health point of view; among the 119 diseases listed, only 31 are zoonotic to one degree or another [12]. The declared priorities of international bodies (WHO, FAO, OIE) federated under the GLEWS [13] programme (*Global Early Warning and Response System for Major Animal Diseases, including Zoonoses*) for the surveillance and monitoring of animal diseases nevertheless derive from an approach first initiated by WHO that gave priority to zoonotic diseases. This is why the GLEWS list includes 6 non-zoonotic and 19 zoonotic diseases.

On the basis of vaccine production, it should be noted that almost all those used in the field of animal health protect against strictly animal pathogens. The rabies vaccine is one of the rare veterinary vaccines meant to protect humans. Certain other veterinary vaccines, such as for leptospira, target a zoonotic agent but are used mainly to protect pets, the exception being the New Zealand cattle vaccination programme that also aims to protect farmers; vaccines against zoonotic agents generally are not meant to protect animals in the name of public health.

#### 2.3.3. Issues at stake in animal health research

**Figure 1:**
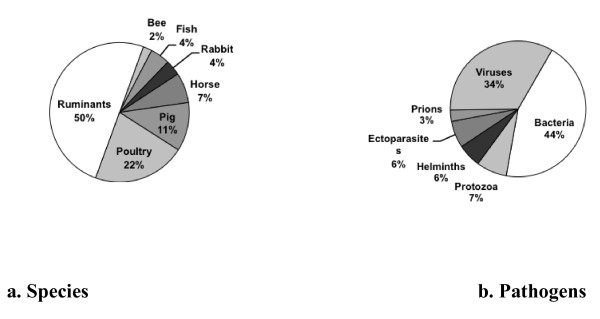
**Distribution of publications on infectious and parasitic diseases in animal health according to the livestock species (a) and pathogens (b) involved**. Analysis in the framework of the European Star-Idaz project [15] of 28 750 international scientific articles published on the subject from 2006 to June 2010.

Precise light was thrown on the subject by a **bibliometric study covering the 2006-2009 period **conducted under the European Era-Net EMIDA programme (Emerging Infectious Diseases of Animals) [14] which focused on infectious and parasitic diseases of production animals. The map generated by the study shows that animal health is situated at the intersection of other disciplinary fields such as human health, but also the health of wild animals and ecosystems, animal nutrition, animal genetics, and animal welfare. The study also demonstrates that barely 20% of the 12 000 publications on infectious diseases surveyed address zoonoses and food safety, and thus have a direct link to public health issues. This means that, in contrast, 80% of the publications address exclusively animal diseases presenting primarily economic, environmental, and animal welfare challenges. The distribution of research work on infectious and parasitic diseases at the international scale [15] according to the production animal species and pathogens involved is presented in Figure 1.

At the European level, it should be noted that an effort to prioritize issues at stake and research involving over 50 infectious and parasitic animal diseases is led by a group of experts under the aegis of Discontools (Disease Control Tools) working with the European ETPGAH platform (European Technology Platform for Global Animal Health). The first outputs may be accessed online on the Discontools web site [16].

Given the breadth of the challenges related to animal health, numerous research questions need to be explored that touch upon different domains of biology and social sciences to broaden existing knowledge, with a continuum from basic to applied research. The questions involve knowledge of pathogens, the relationship between a host (infected animal) and a pathogenic agent, as well as the interaction of pathogens and hosts at the scale of animal populations. The research to be carried out thus aims to propose tools to control the exposure of domestic animals to pathogens, reinforce the resistance of hosts to pathogenic agents (notably through vaccination), and to treat sick animals. The containment and control of diseases through control and prevention programmes also requires assessments of economic and social impacts of health management plans.

In addition to such targetted research, there is a need for fundamental research geared to producing generic knowledge on animal models. The research undertaken in this field is enriching understanding of biology thanks to comparative biology. The diversity of the model species studied, the availability of experimental mechanisms and of biological material, as well as the mastery of particular infectious models, are all important assets for this research, which produces knowledge on living organisms that does not necessarily have an immediate application, but which may prove to be very useful in the future (example of innate immunity molecules-defensins, Toll receptors-identified in invertebrates that have vaccinal and immunomodulatory applications in humans and domestic animals).

## 3. Special features of animal health research

Animal health research is distinguished by particular objectives, methods, biological models and scientific questions. However, there nevertheless are areas of generic and methodological convergence with biomedical research.

### 3.1. Distinguishing features of the objectives, methods, and biological models

First of all, livestock farming is an economic activity whose end goal is to generate revenue. In this context, animal health is one of several factors that farmers must manage; they do so by minimizing their herds' exposure to health risks and by finding the least expensive way to limit the impact of disease [17]. In a given livestock system, diseases are closely linked to the way livestock are managed, notably to parameters related to the quality of housing, nutrition, hygiene, and to animal production levels. The intensification of livestock systems that has taken place in agriculture over the past fifty years has accentuated the tension between limiting inputs, increasing production, and the risk of disease.

Over time, questions regarding livestock health have moved beyond a sole objective of achieving economic gains by reducing disease frequency to addressing the sanitary quality of products of animal origin, reducing the use of xenobiotics, and animal welfare in the interest of public health and sustainable development. The multiplicity of the challenges leads to the question of how the best balance may be achieved between these different parameters. To continue working in this direction, animal health stakeholders, whether from the perspective of research or development, need to establish close ties with livestock sciences and agricultural professionals.

To take into account these elements, population medicine on farms will be needed, as well as research on diseases that specifically recognizes the close connections between health and animal production science. This implies in-depth collaboration with other animal science disciplines on one hand, and with the various stakeholders in the livestock world on the other. The only pertinent research is that carried out in close contact with the actual practices of farmers and animal sectors. For example, within an integrated agriculture framework, integrated research on livestock health management implies solid understanding of the livestock world, requires close collaboration between animal production, genetics, livestock economics, sociology and animal health disciplines, and relies on a partnership with livestock health stakeholders.

A second distinguishing feature of animal health research is the overwhelming predominance of infectious and parasitic diseases, at least for livestock, with a very large diversity of pathologies and a very large repertoire of pathogens involved [18]. Animal health research teams consequently are obliged to study a wide variety of pathogen families, developing in the process a pool of rare and precious skills in virology, bacteriology, parasitology, and medical entomology.

A third distinguishing feature of animal health research is related to the special genetic features of livestock animals. The evolution of animal species, which results in the diversity of species, takes much longer time than phases of domestication, which result in the diversity of breeds. The intensive selection practices implemented over the past fifty years has improved production considerably, but the cost has been a sharp drop in genetic diversity among livestock [19,20]. A distinguishing feature of livestock systems effectively is the possibility of human intervention to select animals for particular genetic traits, most often production (for example, quantity of milk) but also resistance to disease (for example, against scrapie). To understand the genetic foundations of susceptibility to infectious diseases, the duration of co-evolution, genetic diversity, and the respective evolutionary dynamics of hosts and pathogens therefore must be taken into account. The genetic improvement of the immune response is a complex selection objective. It generally either is directed against a single target (pathogen) that is constantly evolving (due to its rapid evolutionary dynamic), or seeks a better overall immunocompetence; in either case, there tends to a negative correlation with the selection of production traits.

Animals of economic importance include species that belong to very distinct animal clades such as fish, bees, chicken, pigs, goats, sheep and cattle. These clades diverged from each other hundreds of millions of years ago. Even within mammals, the Laurasiatheria superorder, which includes ruminants and pigs, and the Euarchontoglires superorder, which includes humans and mice, diverged from each other around 100 million years ago, rendering mice and human phylogenetically closer to each other (so called supra-primates) than they are to ruminants and pigs [21]. These millions of years of separated evolution generated specific anatomical, metabolic and physiological traits, as well as specific commensal-host and pathogen-host relationships. For example, fish show particularities linked to their aquatic environment with some pathogens entering via fins [22]; they present a more primitive immune system and their cells are highly permissive to DNA transfer, allowing highly efficient DNA vaccination [23].

Whereas the basic structures and the generation mechanisms of the T cell receptors and immunoglobulins are similar from teleost fish to higher mammals, each species presents particularities, such as specific isotypes (unlike humans, mice do not secrete IgD or IgG4) and specific mechanisms of antibody diversity generation (gene conversion in chicken, hyper somatic mutations in human and mice). Notably, cytokines are specific to some species; for example, those controlling the production of type I IFN in humans and probably pigs does not exist in mice. Across species, mother to offspring transmission of pathogens and of immunity is strongly dependent on developmental characteristics related to oviparity and variations in placentation modalities. Thus whereas baby mice acquire their immunoglobulin pool during pregnancy by translocation through the placenta, ruminants acquire their immunoglobulin pool at birth via the colostrum due to the relative impermeability of their placenta.

Most basic and applied research is conducted on laboratory mice, in which some human and domestic animal diseases have been experimentally adapted. In many instances, therapeutic and prophylactic treatments that are effective in laboratory mice do no work when transposed to human and veterinary species. This lack of transposition can be explained by the specific physiological traits mentioned above and by the artificial pathological mouse models used in the laboratories. It is very important for pathogen-host interactions and novel therapeutic and prophylactic treatments to be evaluated on the targeted veterinary species, thereby studying the effect in the actual host and consequently limiting a "mouse" bias as much as possible. Research and experiments on "target" species (fish, chicken, pigs, ruminants) therefore often is necessary, and presents an advantage because the research findings may be applied directly to the species without the extra step of validating an extrapolation based on an animal model, in contrast to research undertaken for biomedical applications.

Lastly, there are special features related to the types of actions taken for animal disease control and health management. Beyond vaccination and the protection of livestock, animal health rules covering contagious diseases include a range of control methods, including at times the slaughter of animals to eliminate those posing a risk for unaffected animals and humans. These practices lead to specific research questions regarding intervention mechanisms. At the top of this list is the need to update serological tools so that vaccinated animals may be distinguished from infected animals because disease control measures are different for these two categories of animals. Another priority is the set of questions regarding the comparative economic advantage of different control methods and the conditions by which they are appropriated by livestock farmers and public officials.

While the livestock world has many other distinguishing characteristics, these do not seem to have a notable impact on the manner by which animal health research is conducted.

### 3.2. Special features of scientific questioning

In addition to the aspects discussed in the preceding sections, one of the main distinguishing features of animal health research are the scientific questions pursued, which are posed from the perspective of animal, and not human, health. Consequently, even in the case of zoonotic agents, the questions asked by animal health teams are not the same as those asked by biomedical teams. In the case of zoonotic vector agents, for example, *Bartonella *or *Borrelia *agents of Lyme disease, animal health research would focus on the role of animals as reservoirs of agents potentially pathogenic for humans, and on the elements that allow the development of an infectious agent in its host reservoir versus a human. Biomedical research, on the other hand, would focus on the development of an infectious agent in a human. For prion diseases, an animal health perspective leads to studying the diversity of strains found in the animal and to an attempt to decipher the interactions between the infectious strain and the host species. More broadly, studies of pathogenic agent/host interactions that are pursued from an animal health angle often prove to be fruitful from both a pure and applied perspective. This is due in particular to the genetic knowledge generated on the infected host and the possibility of implementing protocols with an experimental cohort with a defined genetic status. This is, for example, the case with the demonstration in sheep of the modulation of susceptibility to scrapie in connection with the polymorphism of the protein prion coding gene [24].

It thus would appear that, while working on the same agents and with the same tools, the questions pursued in animal health may be different from, and complementary to, those in human biology, and lead to the production of complementary knowledge. It follows that opportunities for collaboration between animal health and biomedical teams should be pursued, each having, through the questions they pursue and their "natural" partnership networks (hospitals versus farms or the environment), access to different and complementary types of samples. For example, collaboration could focus on comparing, with an epidemiological objective, *Bartonella *strains sampled from humans and different animal species.

### 3.3. Generic and methodological areas of convergence with human health

In certain fields, research carried out in human biology and animal health use similar tools, and even the same models, to address research questions. When this is the case, notably in the framework of the study of zoonotic pathogens, the only difference lies in the nature of the questions explored.

In certain circumstances, the convergence continues up to point where the biomedical and animal health teams share the same questions, and then no evident distinguishing feature remains. The development of projects initially focused on animal health progressively may lead the teams involved to pose questions that are increasingly focussed on models shared with human biology. As an illustration, we may cite fundamental research approaches to the molecular mechanisms of the invasion of cells targeted by the influenza virus, or the biological origin of prions and the determinants of the species barrier modulating their transmission capacity. In such cases, it is easy to imagine that the same research could be conducted in research laboratories unrelated to animal health. However, an animal health perspective offers certain advantages, notably expertise for extensive experimental research in a confinement area, and special links maintained through collaborations with other scientists working notably in the fields of pathogenesis and animal genetics.

The discussion presented here was conducted in relation to human biology research work. A parallel approach could be envisioned in relation to work carried out on plant health. Such an analysis may elicit a certain community of tools and methods with animal health, an advantage of comparative biology, but apparently few shared issues at stake for the pathogens of interest.

## 4. Relationships between animal health and human health research

### 4.1. Domestic animal models for human targeted research

Mice often prove to be an inadequate model in physiopathological, prophylactic, and therapeutic studies for humans. This is due to the reduced size of the species, physiological considerations, and the absence of a natural corresponding pathology. With regard to the latter point, it often is necessary to infect a mouse with the human pathogen agent, and thereby create an artificial model without pertinent symptoms. In certain situations, domestic species prove to be better study models for human-oriented research. Domestic species can be infected by viruses that have co-evolved with their host. These diseases present similarities in molecular and physiopathologic mechanisms to human disorders without being zoonotic. Pigs infected by an influenza virus that has adapted to pigs thus suffer an influenza syndrome resembling that found in humans infected with a human influenza virus. Young calves infected by a respiratory syncytial virus distinct from the human virus develop a broncho-pulmonary pathology close to that of a child. These animal disorders thus allow the development of therapeutic, vaccination, and diagnostic strategies that can be adapted or extrapolated to humans.

Furthermore, through evolutionary convergence, certain domestic species present more functional similarities to humans than mice: for example, sheep for respiratory pathology (immunologic study of asthma treatment), and pigs for skin structure (study of transcutaneous therapy or vaccination), cardio-vascular diseases, and the development of spontaneous melanoma where the progression of tumors resembles that observed in humans.

Lastly, domestic animals, due to their large size, allow immune functions to be studied in an original manner that would not be possible with mice. It thus is possible to catheterize lymphatic vessels in pigs, cows, and sheep to study baseline migrant leukocyte populations directly in the lymph during an infection or vaccination, enabling certain immune response features to be monitored in real time.

For these different reasons, in-depth knowledge of domestic animal physiopathologies and the existence of high performance animal experimentation platforms are useful for biomedical research. Overall, the diversity of models (animal species) studied, the foundation of comparative biology, is important to produce general knowledge that can have diverse applications, notably in human biology.

### 4.2. Funding and evaluation of research

Compared to research on pathogens affecting public health, it is notoriously difficult to find funding for research dedicated to animal health that is focussed on non-zoonotic pathogens or to publish the results in high quality scientific journals. These difficulties seem to be inversely proportional to the genericity of the knowledge produced and to its potential biomedical contribution. For example, in a call for proposals on infectious disease research, an excellent project on a non-zoonotic pathogen will systematically be eclipsed by a project addressing a topic such as hemorrhagic fevers due to the evaluators' perception of the stakes involved. Similarily, numerous human health and scientific journals that have a high impact factor due to the larger size of the scientific community involved in human biology compared to animal health, rarely accept an article on non-zoonotic agents that effectively fall outside their domain.

This state of affairs is extremely important to take into consideration given the current imperative to obtain credit to finance research and the use of the "impact factor" criteria in the scientific evaluation of research teams. This point is even more critical as the apparent proximity of animal health and human biology sectors nevertheless does not render their objectives equivalent. An overly hasty approach to the question by evaluators who are ill-informed or insufficiently aware of the issues involved will lead them to apply criteria and indicators to animal health research that are appropriated from human biology and which are completely unsuitable, and indeed unfair, in the field of animal health. Research units that address both zoonotic and non-zoonotic pathogens face a delicate situation. Teams within the same unit are not in the same boat with regard to seeking funding and publication levels.

What emerges from this analysis is that, when research of equivalent scientific quality are considered together, work on non-zoonotic diseases are financed less easily, and are published in journals with a lower impact factor, than work on zoonotic animal diseases. In a similar fashion, research on animal diseases are financed and published less easily than human biomedical research. In the absence of specific corrective action, the existence of a "species barrier" in terms of funding and publication is endangering 80% of animal health research. It thus is absolutely necessary to act far upstream of national and international research programmes by ensuring that calls for research proposals specifically mention the issues at stake in animal health on one hand, and that research organizations for their part officially adopt a policy to recognize the stakes and scientific outputs that are specifically linked to animal health.

### 4.3. Parallels between research, surveillance of diseases and the pharmaceutical industry

#### 4.3.1. Surveillance and control of diseases

A parallel may be drawn between the domain of research and that of disease surveillance and control. OIE officials call attention to a school of thought circulating at the international level that suggests economies of scale would be possible if veterinary medicine services were regrouped with human health facilities in each country. Along the same lines, public services such as disease surveillance are perceived to be expendable variables that may be played with to cut costs in debt-ridden countries. In the same spirit, this school of thought also advocates that only animal diseases posing risks to humans should be considered important due to their zoonotic character. In such a logic of cost-cutting and the regrouping of animal and human health spheres, financial trade-offs naturally would favour human health priorities at the expense of veterinary services.

The OIE's strategy is to take the opposing view which holds that prevention costs less than resolving crises, and that quality prevention is based on national animal health systems that can ensure appropriate surveillance, early detection, transparency, and rapid response to animal disease outbreaks and on a durable network of veterinary services endowed with a specific budget. Thus in 2006, the OIE reiterated its affirmation that veterinary services were a global public good [25]. The disastrous consequences of cutbacks in public services, and the efficacy of the preventative and global approach taken by the OIE, is leading progressively to a swing of opinion in favour of this approach. This change is visible, for example, in the international documents debated during successive forums on the control of avian influenza [26].

#### 4.3.2. Pharmaceutic industry

Most pharmaceutical companies have subsidiaries dedicated to animal health, which is related to the fact that economic scales between animal and human health cannot be compared; as an example, sales of a human vaccine may be 20 to 50 times higher than those of a veterinary vaccine. If a choice must be made between two very different vaccine projects, even if each is a priori profitable, the human vaccine automatically will be chosen over the veterinary vaccine. In the same manner, shared services will be put at the disposal of the human vaccine project given the higher economic stakes involved. Lastly, it also is more difficult to find public funding, and thus complementary private funding, for the development of vaccines against non-zoonotic pathogens than for human vaccines. A fusion between human and animal activities would translate into the disappearance of the animal sector, or into animal models being developed only when they have a direct interest for humans. In contrast, what is shared by animal and human vaccines is an ensemble of vaccine production technology, innovations in this field and preceding research on pathogen families, cytology, certain features of immunology, all knowledge that deserves to be shared between human and animal health in the form of cooperation.

### 4.4. The "One World, One Health" approach

As mentioned by the Director of the OIE in an editorial [27], the "One World, One Health" approach is indispensable in the sense that "*the only way to prevent all these new hazards (zoonotics) is to adapt the existing systems of health governance at world, regional and national levels in a harmonised and coordinated manner*", but "*the concept "One World, One Health" should not serve as a pretext for dangerous initiatives like trying to achieve economies of scale based on purely theoretical notions worthy of a sorcerer's apprentice, such as trying to merge the Veterinary Services and the Public Health Services*". Taking this perspective, it effectively is out of the question to merge services because each must assume its functions with the resources dedicated to it and the approaches suited to its particular mission; however, it is necessary to develop collaboration, cooperation, and synergies [28]. For the past few years, there has been a concensus on this issue among the OIE, FAO, and WHO. Different discussions are underway to define ways to implement this cooperation between organisations.

## 5. Conclusion

The present discussion, the opinion of experts, and a critical reading of the literature has led to the following observations.

International bodies (WHO, FAO, OIE) affirm that, over and above the threat of diseases that can be transmitted to humans (zoonotic diseases), the **challenges facing the field of animal health **are considerable. They concern food security, economics, agriculture and associated economic activities in both industrialized and developing countries. The challenges facing animal health, beyond those posed by zoonotic diseases, overlap with those of public health and the environment, notably regarding the use of xenobiotics and the development of antibiotic resistance.

The **distinguishing features of animal health research **are methodological and scientific in nature. They notably pertain to special biological features of domestic species and to the interaction between humans in their practice of livestock husbandry and animals in their biology and evolution. Animal biology generally does not pursue the same scientific questions as human biology, even when the same pathogens are being studied, and the discipline is rooted in a very specific agricultural and economic context. For animal health stakeholders, whether from the perspective of research or development, finding an optimal balance between the economic profitability of a farm, animal welfare, the maintenance of animal health and the quality of products of animal origin involves close collaboration between animal husbandry sciences and the agricultural profession.

**Knowledge produced by comparative biology **is fed by research conducted on animal species. For example, animal models are a source of generic knowledge due to their special evolutionary features and, in certain cases, their functional similarities with humans. The diversity of the model species studied and the control of particular infectious diseases contribute greatly to the production of knowledge about living organisms.

These observations present a strong case in favor of taking into account the uniqueness of animal health research, in terms of its organization, evaluation, and funding, compared to biomedical research. If this is not done, strictly biomedical priorities will lead to the elimination, sooner or later, of quality research on non-zoonotic animal diseases. A special "treatment" of this research thus is necessary with regard to the issues at stake; specially designed calls for proposals should be dedicated to the field, the field's journal corpus should be recognized as being different from that of biomedical research, and the research should be evaluated in the light of this specific corpus.

The "One Health" approach is important insofar as it argues that the management of health requires reinforced coordination between human and animal components and, in the same manner, in-depth collaboration between biomedical and animal health research. The organization of such collaboration can only reinforce the capacity of both groups to produce relevant science, and to realize the potential of research efforts and more global approaches integrating human and animal components in federated projects.

In terms of research, this collaboration may assume different forms and take place at different levels, ranging from cooperation between teams up to the organization of research and its funding. The questions explored in animal health and human biology regarding the same zoonotic pathogen frequently are complementary. They allow scientific collaborations to be built that can respond to more general questions, and notably to address the complexity of the biological systems of certain diseases. Another form of collaboration is the establishment of calls for joint public health and animal health proposals for research on pathogens whose study and control require combined research approaches. This has been the case for research on transmissible spongiform encephalopathies, with joint animal-human calls for projects and pluridisciplinary projects in the United Kingdom, Netherlands, Germany, France and the European Union. At a more general level, comparative biology represents a precious source of knowledge.

## Competing interests

The authors declare that they have no competing interests.

## Authors' contributions

All authors participated in the collective discussion on the special issues of animal health research, search for bibliography and participated in the writing of the paper in their field of competence; more precisely, VB, JLG, PR, ISC, MVT, SZ and EZ were involved in the field of microbiology, ISC in immunology, CF and CD in epidemiology, BB in genetics, JBC and EZ in animal sciences, SK in economics, DT in sociology. CD, CF and SK were involved in the discussion with scientists from OIE, CD, CF and SZ with Société Merial, ISC, SZ and MVT with Institut Pasteur. TP and CD designed the work and defined the working group. CD chaired the discussions and coordinated the paper. All authors read and approved the final manuscript.
